# Bone Coverage around Hydroxyapatite/Poly(_L_-Lactide) Composite Is Determined According to Depth from Cortical Bone Surface in Rabbits

**DOI:** 10.3390/ma14061458

**Published:** 2021-03-17

**Authors:** Kazuaki Morizane, Koji Goto, Toshiyuki Kawai, Shunsuke Fujibayashi, Bungo Otsuki, Takayoshi Shimizu, Shuichi Matsuda

**Affiliations:** The Department of Orthopaedic Surgery, Graduate School of Medicine, Kyoto University, 54 Shogoin-Kawaharacho, Sakyo-ku, Kyoto 602-8570, Japan; morizane@kuhp.kyoto-u.ac.jp (K.M.); kawait@kuhp.kyoto-u.ac.jp (T.K.); shfuji@kuhp.kyoto-u.ac.jp (S.F.); bungo@kuhp.kyoto-u.ac.jp (B.O.); takayosh@kuhp.kyoto-u.ac.jp (T.S.); smat522@kuhp.kyoto-u.ac.jp (S.M.)

**Keywords:** biodegradable, bioactive, poly(_L_-lactide), composite, foreign body reaction

## Abstract

Composites of unsintered hydroxyapatite (HA) and poly(_L_-lactide) (PLLA) reinforced by compression forging are biodegradable, bioactive, and have ultrahigh strength. However, foreign body reactions to PLLA and physical irritation can occur when not covered by bone. We aimed to confirm the relationships between the depth of the implanted HA-PLLA threaded pins and the new bone formation. We inserted HA-PLLA composite threaded pins (diameter: 2.0 or 4.5 mm) into the femoral and tibial bones of 32 mature male Japanese white rabbits (weight 3.0–3.5 kg) with the pin head 1 or 0 mm below or protruding 1 or 2 mm above surrounding cortical bone. Eight euthanized rabbits were radiologically and histologically assessed at various intervals after implantation. Bone bridging was complete over pins of both diameters at ~12 weeks, when inserted 1 mm below the surface, but the coverage of the pins inserted at 0 mm varied. Bone was not formed when the pins protruded >1 mm from the bone surface. No inflammation developed around the pins by 25 weeks. However, foreign body reactions might develop if composites are fixed above the bone surface, and intraosseous fixation would be desirable using double-threaded screws or a countersink to avoid screw head protrusion.

## 1. Introduction

Various materials can be used for bone fixation. Bioabsorbable bone fixation devices have become prevalent in clinical practice. Specifically, composites of unsintered hydroxyapatite (HA) and poly(_L_-lactide) (PLLA) reinforced by compression forging are biodegradable, bioactive, and have ultrahigh strength [1,2]. They are available for clinical practice as Super-Fixsorb or Osteotrans Plus (Teijin Medical Technologies Co., Ltd., Osaka, Japan). HA-PLLA composites have some advantages compared with other implants. First, their mechanical properties are comparable with those of the human cortical bone [1,3]. HA-PLLA composites, which were implanted in the subcutis of the rabbits, could maintain higher bending strength than human cortical bone for more than 25 weeks after implantation in rabbits [1,4]. Second, HA-PLLA composites are completely replaced by the surrounding bone, and there is no need to remove them after the bone has healed [2,5,6,7]. 

However, complete degradation and resorption of HA-PLLA composite rods (3.2 mm in diameter) implanted in rabbit femora were reported to need several years [2,5]. Then, concerns were raised about foreign body reactions to the extraosseous part of the HA-PLLA, which could cause irritation or discomfort in clinical practice. Landes et al. reported clinical outcomes after the internal fixation of malar and midfacial fractures using HA-PLLA plates and screws, and 2 of 29 patients had foreign body reactions that were treated by removing the implants [8].

To decrease the risk of the foreign body reaction, it is desirable for the HA-PLLA screws to be covered with new surrounding bone early after implantation. However, how to achieve this is yet to be known. One solution might be to insert the screw as deeply into the bone as possible. As far as we can ascertain, how bone formation over an HA-PLLA composite is affected by the depth of the screw head has not been investigated. Therefore, we aimed to confirm the relationship between the depth of the HA-PLLA composites and the new surrounding bone formation in vivo.

## 2. Materials and Methods

### 2.1. Material Preparation

**Figure 1:**
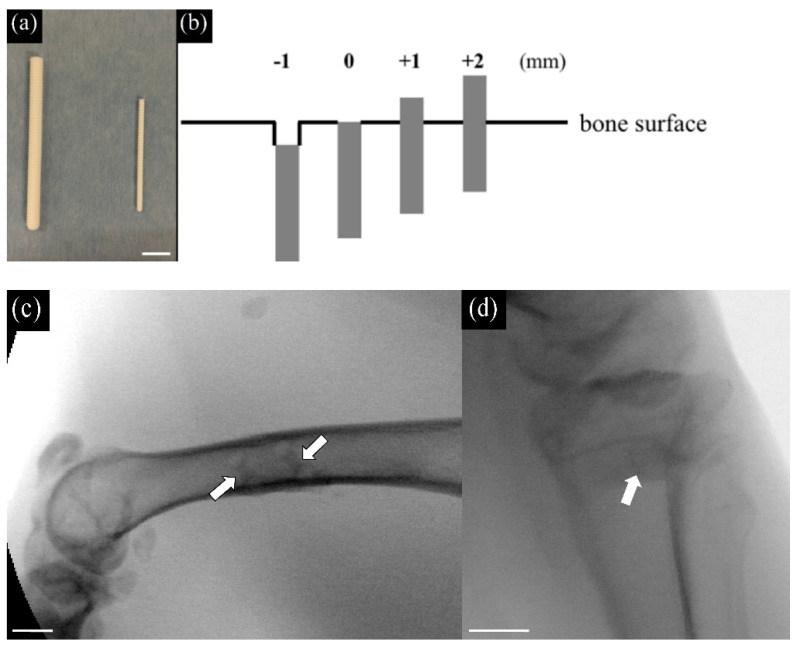
HA-PLLA threaded pins, their insertion height, and X-ray photographs. Left to right: (**a**) HA-PLLA threaded pins (diameter 4.5 and 2.0 mm). Scale bar: 10 mm. (**b**) Insertion height determined based on bone surface. (**c**) The X-ray photograph of inserting threaded pins of 2.0 mm into the lateral midshaft of the femur. Scale bar: 10 mm. (**d**) The X-ray photograph of inserting a threaded pin of 4.5 mm into the proximal medial tibia. Arrows indicate the inserted pins. Scale bar: 10 mm.

The pins consisted of bioactive, bioresorbable unsintered, and uncalcined HA, with a calcium to phosphate ratio of 1.69. The weight ratios of HA and PLLA were 30% and 70%, respectively, and the HA-PLLA pins were reinforced by compression forging [1]. Specifically, we used HA-PLLA threaded pins of 2.0 mm and 4.5 mm diameter (Super-Fixsorb 30, Teijin Medical Technologies Co., Ltd., Osaka, Japan) (Figure 1a). The length of pins with a diameter of 2 and 4.5 mm were cut to 7 mm, and 10 mm, respectively. 

### 2.2. Surgical Procedure

This study was approved by the Animal Research Committee, Graduate School of Medicine, Kyoto University, Japan, following the guidelines of the National Institutes of Health Guide for Care and Use of Laboratory Animals (approval number: Med Kyo 17531). Thirty-two mature male Japanese white rabbits (weight 3.0–3.5 kg) were assigned to groups (n = 8 each) for analysis at 4, 8, 12, and 25 weeks after inserting pins. The rabbits were anesthetized with an intravenous injection of pentobarbital sodium (40 mg/kg), isoflurane inhalation, and locally administered 0.5% (*w/v*) lidocaine. The HA-PLLA pins were sterilized with ethylene oxide gas. Pins with diameters of 2.0 and 4.5 mm were respectively inserted into the lateral midshaft of the femur, and the bilateral proximal medial tibia under standard aseptic conditions.

Unilateral femoral shafts were exposed using a lateral approach, and two holes (diameter 2.0 mm, at least 1.2 cm apart) were created monocortically using a manual drill on the lateral side of the femur [9]. Bone debris was rinsed with saline, then the 2 mm HA-PLLA pins were inserted into the holes to depths of −1 and 0 mm below, and +1, and +2 mm protruding from the surface (Figure 1b,c) and measured intraoperatively using a ruler.

The proximal medial portions of the bilateral tibial bones were exposed. A 3 cm longitudinal incision was made on the medial side of the knee, and the fascia and periosteum were incised and retracted to expose the tibial cortex. A hole of 4.5 mm diameter and perpendicular to the surface of the tibia was manually drilled. The hole was irrigated with saline, then 4.5 mm HA-PLLA pins diameter were implanted to depths of −1 and 0 mm below, and +1, and +2 mm above the tibial surface (Figure 1d). After irrigation, the fascia and skin were sutured layer by layer. Rabbits were housed individually in standard rabbit cages and fed with standard rabbit food and water.

At 4, 8, 12, and 25 weeks after implantation, eight rabbits were euthanized with an overdose of intravenous pentobarbital sodium, then segments of midshaft femurs and proximal tibiae containing the HA-PLLA pins were excised and kept moist. Four pins at each position in the eight femoral and 16 tibial bones of eight rabbits were assessed at each time point, and all femoral and tibial bones were radiologically and histologically analyzed.

### 2.3. Radiological Analyses

**Figure 2:**
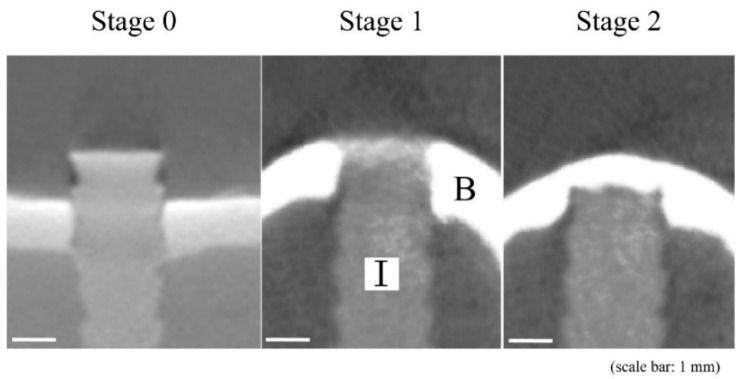
Radiological classification of bone bridging above implant by modified Goldberg classification [10]. The stage of 0 indicated no bone bridging, 1 indicated possible bone bridging, and 2 indicated complete bone bridging above the implant. B, bone; I, HA-PLLA threaded pin.

Slices (thickness, 0.04 mm) of all specimens were analyzed using an SMX-100CT-SV-3 Micro-CT system (Shimadzu Corp., Kyoto, Japan). Three-dimensional images of the harvested bone including the HA-PLLA pins were reconstructed using VG studio MAX 2.2 software (Volume Graphics GmbH, Heidelberg, Germany). We evaluated new bone formation above the implanted pins using a modification of the Goldberg classification (Figure 2) [10].

### 2.4. Histological Analyses

**Figure 3:**
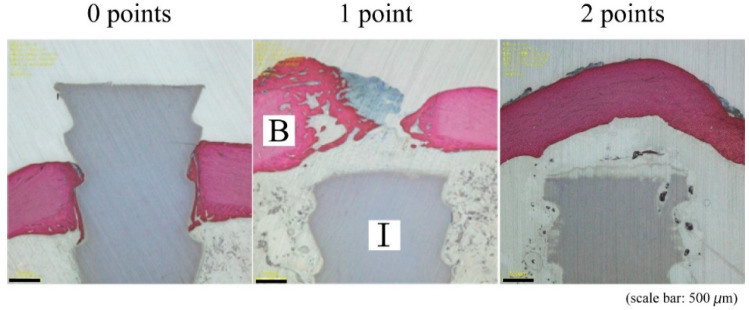
Histological scores for bone bridging above implant by modified Garcia scoring [12]. The point of 0 indicated no bone bridging or only fibrous tissue bridging, 1 indicated cartilage or slight bone bridging, and 2 indicated complete bone bridging above the implant. B, bone; I, HA-PLLA threaded pin.

Specimens were fixed after micro-CT analyses in phosphate-buffered 10% formalin for 10 days, dehydrated in a graded series of 70%, 80%, 90%, 99%, and 100% (*v/v*) ethanol for 3 days at each concentration, and embedded in polyester resin. Sections (500 μm) were cut perpendicular to the bone axis and parallel to the implant axis using a BS-3000CP band saw (Exakt Apparatebau GmbH, Germany), then ground to a thickness of 60–80 µm using an MG-4000 Micro-grinder (Exakt Apparatebau GmbH). Soft tissues and calcified bone in each section were stained with Stevenel blue and van Gieson picrofuchsin (bright red), respectively [11]. Histological sections were analyzed using an Eclipse 80i light microscope (Nikon, Tokyo, Japan) with a DS-55M-L1 digital camera (Nikon), and modified Garcia scoring (Figure 3) [12].

## 3. Results

### 3.1. Radiological Results

**Figure 4:**
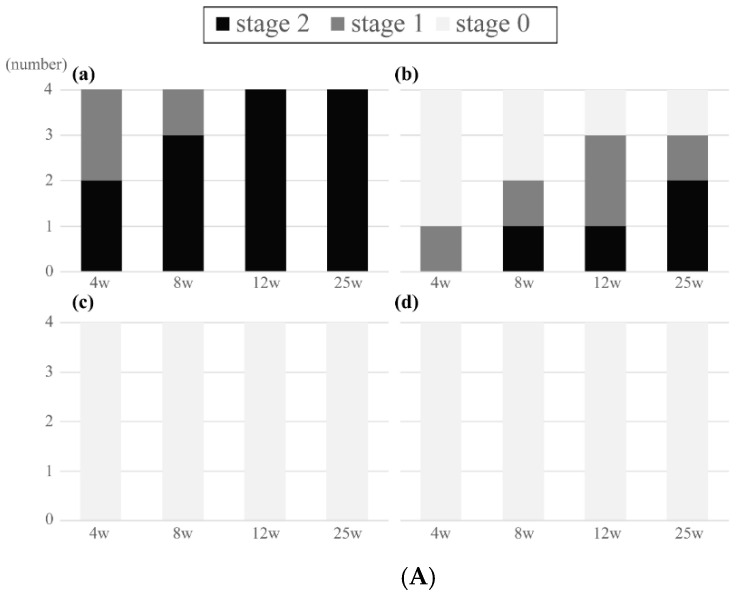

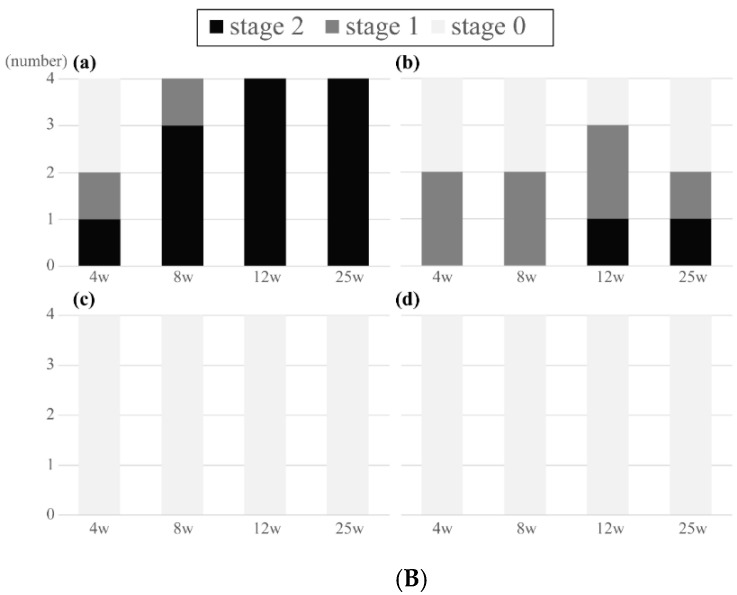
Radiological results of bone bridging above the threaded pins of (**A**) 2.0 mm and (**B**) 4.5 mm diameter. (**A**) Diameter: 2.0 mm. Depth: 1 (a) and 0 (b) mm. Protrusion: 1 (c) and 2 (d) mm. (a) Bone bridging was complete above all implants by 12 weeks. (b) At 25 weeks, two pins were completely covered by the new bone, and one had possible bone bridging. (c, d) Bone bridging was absent at all time points. (**B**) Diameter 4.5 mm. Depth: 1 (a) and 0 (b) mm. Protrusion: 1 (c) and 2 (d) mm. (a) Bone bridging was complete above all implants by 12 weeks. (b) Two had possible bone bridging at 4 and 8 weeks. At 12 weeks, one pin was completely covered by the new bone, and two had possible bone bridging. (c, d) Bone bridging was absent at all time points.

Figure 4A shows the radiological results of the HA-PLLA pins of 2.0 mm diameter.

New bone bridged above three of four pins implanted at a depth of 1 mm within four weeks. Bone bridges were complete above all implants by 12 and 25 weeks. Possible bone bridging was identified in one of four pins implanted at 0 cm, whereas no bone formation was observed in the other three pins at 4 weeks. At 25 weeks, two pins were completely covered by the new bone, and one each had possible and no bone formation. Bone bridging was absent at all time points when the pins protruded 1 and 2 mm above the surface.

Figure 4B shows the results of the HA-PLLA pins with a diameter of 4.5 mm. Bone bridging was complete in all pins that were implanted at a depth of 1 mm at 12 and 25 weeks. Overall, new bone formation tended to be similar between the pins with diameters of 2.0 and 4.5 mm.

### 3.2. Histological Analyses

**Figure 5:**
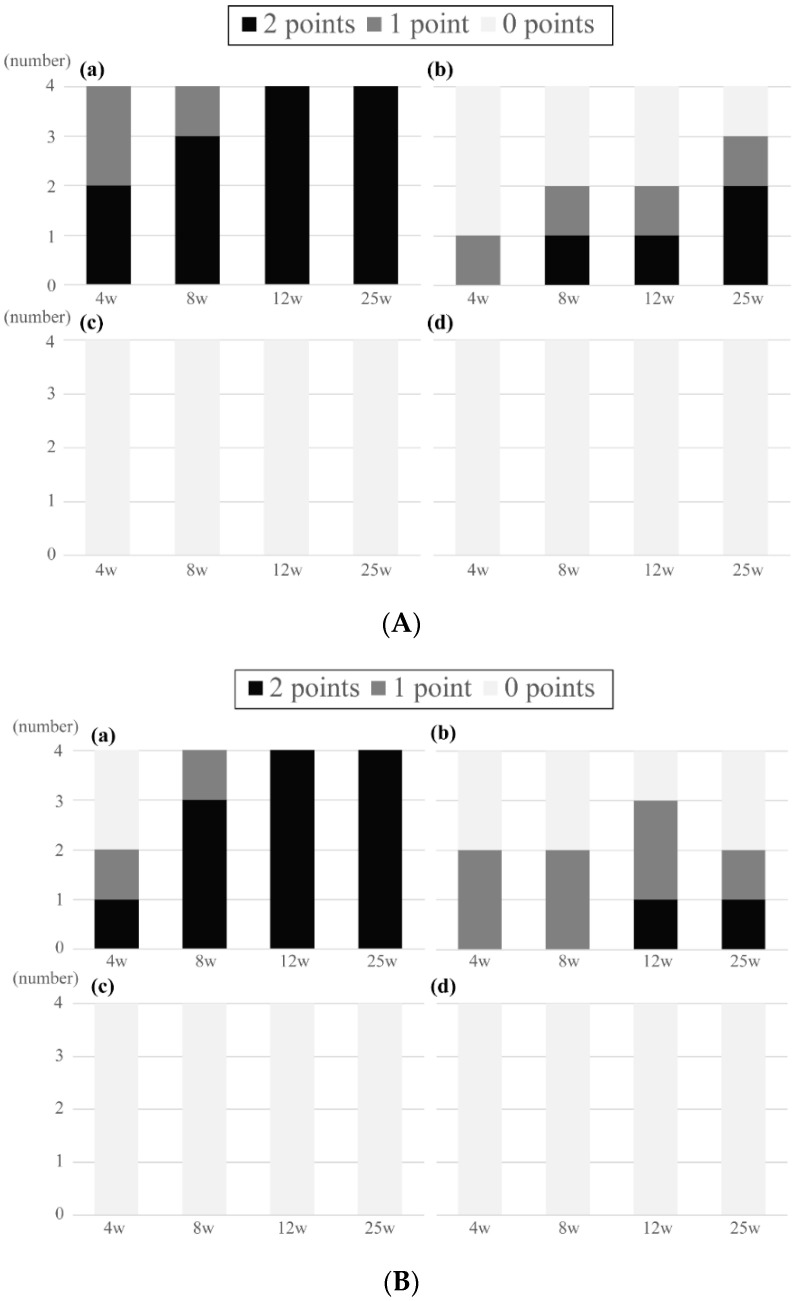
Histological results of bone bridging above the threaded pins of (**A**) 2.0 mm and (**B**) 4.5 mm diameter. (**A**) Diameter 2.0 mm. Depth: 1 (a) and 0 (b) mm. Protrusion: 1 (c) and 2 (d) mm. (a) Bone bridging was complete above all implants by 12 weeks. (b) At 25 weeks, two pins were completely covered by the new bone, and one had possible bone bridging. (c, d) Bone bridging was absent at all time points. (**B**) Diameter 4.5 mm. Depth: 1 (a) and 0 (b) mm. Protrusion: 1 (c) and 2 (d) mm. (a) Bone bridging was complete above all implants by 12 weeks. (b) At 12 weeks, one pin was completely covered by the new bone, and two had possible bone bridging. (c, d) Bone bridging was absent at all time points.

When taking out the specimen from the euthanized rabbits, no apparent swelling or redness around the specimen was observed macroscopically, and no evidence of loosening of the inserted pins was detected. Figure 5A,B show the histological results of the HA-PLLA pins with diameters of 2.0 and 4.5 mm. All histological scores corresponded with radiological stages at 12 weeks, except for one implanted pin with a diameter 2.0 mm. Fibrous tissue could not be differentiated from the cartilage above the implant clearly, and the score of 1 in the modified Garcia classification was only determined when partial bone bridging was detected.

**Figure 6:**
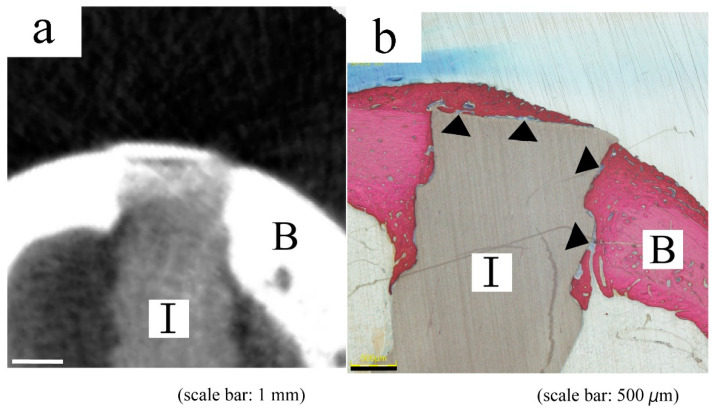
Typical micro-CT (**a**) and histological (**b**) images of same pin with 2.0 mm diameter at 12 weeks. Both images indicate incomplete bone bridging. B, bone; I, HA-PLLA threaded pin.

Histological staining detected new bone formation along the implant more clearly than micro-CT imaging and revealed direct contact with some intervening soft tissues between the implant and bone (Figure 6). Soft tissues surrounding the composite pins were not apparently inflamed.

## 4. Discussion

Composite HA-PLLA pins have been widely applied in orthopedic, craniofacial, oral, maxillofacial, plastic, and reconstructive surgeries since 2003. The effectiveness and advantages of HA-PLLA composites have been established [13,14,15,16,17,18,19,20]. However, a few case reports have described complications [8,21,22,23] such as foreign body reactions and broken screw heads caused by foreign body reactions in surrounding soft tissue. Moreover, these complications developed around one year postoperatively. Long-term complications of foreign body reactions can be attributed to slow degradation of the HA-PLLA composites. To our knowledge, intraosseous complications of the HA-PLLA composites have not been reported. The foreign body reaction would not occur if the bony tissues of the cortex that directly contact the HA-PLLA composite could extend and completely cover the surface of the composite. Thus, determining the threshold within which HA-PLLA composites are covered with new surrounding bone after implantation is important to eliminate the foreign body reaction.

Here, we investigated the formation of new bone above composite pins implanted at various depths relative to the surrounding cortical surface. The radiological and histological findings showed new bone formation above the implants. Bone bridging was complete at 12 and 25 weeks when pins with diameters of 2.0 and 4.5 mm were inserted to a depth of 1 mm below the bone surface, respectively. Considering that a pin diameter of 4.5 mm is quite large for rabbit tibiae, the heads of HA-PLLA composite screws with a diameter of 4.5 or 6.5 mm should be completely covered with new bone within 12 weeks when inserted below the surface of human long bones. Coverage varied on pins inserted 0 mm from the cortical surface, and no new surrounding bone formed on pins inserted with protrusions of 1 mm above the bone surface. Therefore, screw heads fixed above the bone surface confer the risk of a foreign body reaction.

Accordingly, the intraosseous implantation of the threaded pins or the development of double-threaded screws could help to avoid foreign body reactions. A countersink would also help to minimize the protrusion of the screw head. However, excessive countersinking damages the cortical bone structure and weakens compression force [24]. Therefore, further investigations should determine the yield strength of countersunk cortical bone fixed with HA-PLLA screws.

New bone formation along the implant and direct contact between the implant and bone were more clearly visualized by histological staining than by micro-CT imaging. This was presumably due to the high density of the HA-PLLA composite in micro-CT images. The similar CT values of bridging bone and composites containing HA particles caused difficulties distinguishing new bone from the implant.

An inflammatory response of soft tissues around the HA-PLLA composite did not arise within 25 weeks macroscopically. In the histological and radiological analyses, the bone tissue around the inserted pins directly contacted them, which also indicated absence of the foreign body reaction. This result was consistent with the reports indicating that postoperative complications related to the foreign body reactions take at least one year to develop [8,21,22,23]. The long-term complications of HA-PLLA composite implants should be investigated. 

This study has several limitations. Because our results were derived from rabbits, they cannot be applied directly to the depth and diameter of implants in human bone. However, the time course of HA-PLLA composite osteointegration in rabbits is consistent with the clinical findings [2,5,6,7]. Therefore, rabbits were deemed appropriate for this study. We did not compare bone coverage over the HA-PLLA composites with other materials such as titanium and stainless steel. Thus, we could not evaluate the effects of HA-PLLA composite bioactivity and biodegradability on bone coverage. We sacrificed the periosteum when inserting the composites. The rabbit periosteum is very thin, vulnerable and could not be repaired by suturing. The periosteum plays an important role in repairing bone after bone injury. However, we could not always repair the periosteum over screw heads in clinical practice. Further studies are necessary to evaluate the effects of retaining periosteum coverage. Finally, we could not differentiate the fibrous tissue from the cartilage above the implant clearly in the histological analyses. Further histological analyses should be considered to clarify the process of bone bridging above the HA-PLLA composites.

## 5. Conclusions

Bone coverage was complete when pins with diameters of 2.0 and 4.5 mm were inserted at a depth of 1 mm below the bone surface within 12 weeks. In contrast, no new surrounding bone formed when the pins protruded >1 mm from the bone surface. The intraosseous implantation of threaded pins or the development of a double-threaded screw might help to avoid foreign body reactions. Otherwise, using a countersink to avoid screw head protrusion is recommended.

## Data Availability

The data presented in this study are available on request from the corresponding author. The data are not publicly available due to the possibility of future patent acquisition.
